# Externalized Conductor Cables in QuickSite Left Ventricular Pacing Lead and Riata Right Ventricular Lead in a Single Patient: A Common Problem With Silicone Insulation

**DOI:** 10.4021/cr179w

**Published:** 2012-09-20

**Authors:** Umashankar Lakshmanadoss, Vera Hackett, Pramod Deshmukh

**Affiliations:** a Division of Cardiology, Guthrie Clinic, Sayre, PA, USA

**Keywords:** Insulation defect, Externalized leads, QuickSite coronary sinus pacing lead, Riata lead

## Abstract

QuickSite (St Jude Medical, Sylmar, CA, USA) is a silicone and polyurethane-insulated coronary sinus pacing lead. Riata lead (St Jude Medical, Sylmar, CA, USA) is a silicone insulated right ventricular shock lead. Recently, insulation breach of silicone based leads raised a huge concern. Fluoroscopic examination of these two leads in the same patient revealed externalization of these two leads. Same mechanism producing insulation breach of Riata lead may be involved in externalization of QuickSite LV lead as distal part of insulation is also made of silicone.

## Introduction

Recently, insulation breach of silicone based leads raised a huge concern [1]. Here we present a case of externalized QuickSite (St Jude Medical, Sylmar, CA, USA) coronary sinus pacing lead which is a silicone and polyurethane-insulated lead and externalized Riata lead (St Jude Medical, Sylmar, CA, USA), which is a silicone based insulated lead.

## Case Report

**Figure 1:**
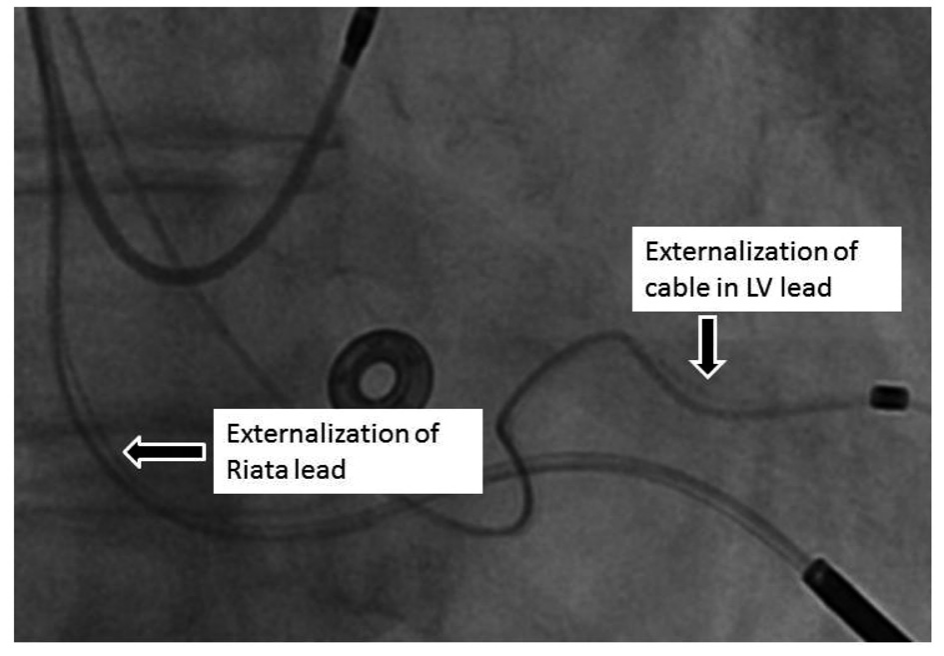
Externalization of the St Jude QuickSite Left Ventricular Pacing lead and Riata RV Shock lead.

A 67-year-old Caucasian female with nonischemic cardiomyopathy (LVEF 20%), wide QRS (140 msecs), NYHA class III symptoms had cardiac resynchronization therapy-defibrillator (CRT-D) implanted for primary prevention of sudden cardiac arrest and management of heart failure in 2006. An Epic HF model V-377 device with St Jude, Riata lead, Model 1581 and QuickSite coronary sinus pacing lead, Model 1056T was implanted. At the time of implantation, all electrical pacing parameters were within acceptable range. Her device reached ERI (Elective replacement indicator) in 2012. At the time of device generator change, again pacing and electrical parameters were within nominal range. High resolution fluoroscopic examination of the leads was done at the time of the device generator change and revealed externalization of distal portion of QuickSite lead and externalization of the Riata lead at the level of the tricuspid ring (Fig. 1) (also see supplementary data video at www.cardiologyres.org). As electrical and pacing parameters were within normal limits, no further intervention was planned. The patient is being followed closely with remote monitoring.

## Discussion

To our knowledge, this is the first report of the externalization of both Riata lead and QuickSite LV lead in a same patient.

Recently there have been multiple reports about fluoroscopic separation of the Riata leads which usually occurred at the distal part of the lead or near the tricuspid valve apparatus [1]. Riata lead (St Jude Medical, Sylmar, CA, USA), is a silicone based insulated lead. QuickSite LV Model 1056T lead is a polyurethane lead body transitioning to a distal silicone tip section for optimal pushability, torque transfer and tip flexibility. It is possible that the same mechanism producing insulation breach of Riata leads may also be involved in externalization of QuickSite LV lead as distal part of the lead is made of silicone material. The inner conductors of QuickSite leads are coated with ethylene tetra fluoro ethylene (ETFE). If ETFE coating is intact, leads may not manifest with change in electrical parameters. However, if ETFE coating is compromised, electrical function of these leads may be compromised leading on to electrical failure [2]. Recently St Jude Medical has discontinued distribution of QuickSite lead based on findings with leads returned to the manufacturer.

### Conclusion

Externalization of the intracardiac leads is a common problem in silicone based insulated leads. Adverse effects of externalized Riata leads are well documented [1]. Adverse effects of these externalized cables of LV leads are unknown at this time. Whether they could lead to pacing failure and hence hastening heart failure or increasing the number of non-responders of cardiac resynchronization therapy, is not known at this time. These patients should be monitored closely, by regular remote transmission and/or by clinical encounter looking for heart failure.
